# Psychophysiological Acute Effects of Functional Neurology Intervention on Vestibulo-Ocular Reflex Dysfunction

**DOI:** 10.3390/jfmk10020146

**Published:** 2025-04-27

**Authors:** Guillermo Escribano-Colmena, Jorge Rey-Mota, Ana Isabel Beltrán-Velasco, Vicente Javier Clemente-Suárez

**Affiliations:** 1 Faculty of Sports Sciences, Universidad Europea de Madrid, Tajo Street, s/n, 28670 Madrid, Spain; escribanocolmenaguillermo@gmail.com (G.E.-C.); jreymota@gmail.com (J.R.-M.); 2 Psychology Department, Facultad de Ciencias de la Vida y la Naturaleza, Universidad Antonio de Nebrija, 28240 Madrid, Spain; 3 Grupo de Investigación en Cultura, Educación y Sociedad, Universidad de la Costa, Barranquilla 080002, Colombia

**Keywords:** functional neurology, vestibulo-ocular reflex, neuromuscular reflex, vestibular dysfunction, neuroplasticity, psychophysiology

## Abstract

**Objectives**: The present study aimed to analyze the psychophysiological and neuromuscular reflex modifications following a single functional neurology intervention in individuals presenting vestibulo-ocular reflex (VOR) cancellation dysfunction. **Methods**: A total of 66 healthy participants, comprising an experimental group (*n* = 48; 22 females, 26 males; mean age 28.1 ± 7.8 years) and a control group (*n* = 18; 9 females, 9 males; mean age 28.6 ± 7.0 years), underwent comprehensive assessments at four distinct measurement moments: baseline, post-indicator muscle failure pre-intervention, immediately post-functional neurology intervention, and post-intervention indicator muscle failure, assessing neuromuscular (handgrip strength) and psychophysiological parameters, including blood oxygen saturation, heart rate, cortical arousal (critical flicker fusion threshold, CFFT), and pain perception (pressure pain threshold, PPT). The functional neurology treatment was tailored based on the ^®^NeuroReEvolution protocol, emphasizing individualized proprioceptive recalibration, trigger point desensitization, and holistic neuroreflex modulation. **Results**: Statistical analyses indicated significant improvements within the experimental group following intervention. Specifically, tolerance to VOR cancellation stimuli significantly increased from a baseline of 1.0 ± 0.0 to 129.0 ± 36.7 post-intervention (*p* < 0.001, η^2^ = 0.926), whereas the control group demonstrated no meaningful change. Furthermore, significant enhancements were noted in pressure pain threshold (27.49 ± 0.67 to 35.69 ± 0.60 kgf; *p* = 0.029), handgrip strength (20.41 ± 0.72 N to 26.56 ± 0.52 N; *p* = 0.012), and critical flicker fusion threshold (32.24 ± 0.45 Hz to 38.32 ± 0.60 Hz; *p* = 0.003). **Conclusions**: The results of this study demonstrate that a single functional neurology intervention significantly improved psychophysiological responses and neuromuscular reflex performance in participants with vestibulo-ocular reflex (VOR) cancellation dysfunction. Specifically, the intervention led to marked enhancements in pain tolerance, cortical arousal, and handgrip strength, and notably, an increased tolerance to VOR cancellation stimuli, indicating improved vestibular control. Cardiovascular parameters remained stable, highlighting the safety of the intervention. These findings support functional neurology as an effective therapeutic approach to address VOR-related dysfunctions by promoting neurophysiological resilience and motor function optimization.

## 1. Introduction

The vestibulo-ocular reflex (VOR) is a fundamental neural mechanism that stabilizes vision during head movements by producing compensatory eye movements in the opposite direction to maintain fixation on a visual target [1]. This reflex is critical for preserving visual acuity during dynamic activities such as walking, running, or turning the head. When the VOR fails or is improperly regulated, individuals may experience blurred vision, dizziness, vertigo, and impaired postural stability, significantly reducing their quality of life [2]. Dysfunction of the VOR not only disrupts ocular motor control but also affects broader neuromuscular and psychophysiological systems. Proper VOR function requires the precise integration of vestibular, proprioceptive, and visual information to maintain postural stability and spatial orientation. Impairments in VOR processing can lead to compensatory overactivation of cortical and neuromuscular systems, increased cognitive load, and altered psychophysiological responses such as changes in arousal and pain perception. A crucial adaptation of the VOR is its cancellation during specific tasks requiring voluntary eye movement tracking of a moving object, such as reading or following a moving ball in sports [3]. VOR cancellation is a complex process involving the suppression of reflexive eye movements through higher-level cortical control, particularly from the cerebellum, parietal cortex, and frontal eye fields [4]. This suppression enables individuals to dissociate head and eye movements when necessary, a capacity that is essential for activities requiring precise visual–motor coordination.

Deficits in VOR cancellation mechanisms can result from various causes, including peripheral vestibular loss, central nervous system lesions, neurodegenerative diseases, or concussive injuries [5,6]. These deficits not only impair gaze stability but can also exacerbate balance disorders and cognitive load, as the central nervous system must allocate additional resources to compensate for impaired sensory integration [7]. Research has demonstrated that individuals with impaired VOR cancellation exhibit greater postural instability and are at increased risk of falls, highlighting the clinical relevance of early detection and targeted intervention [8]. Effective VOR cancellation depends on the efficient integration of multisensory information, primarily vestibular, visual, and proprioceptive inputs, which are processed and modulated by central pathways, including the cerebellum and the vestibular cortical network [9]. Central integration ensures the adaptability and suppression of reflexes when voluntary actions are prioritized. Therefore, therapeutic strategies aimed at improving VOR function must address not only peripheral vestibular inputs but also central sensory processing and motor planning circuits.

Functional neurology presents a promising therapeutic approach to address these dysfunctions by leveraging principles of neuroplasticity and targeted sensory–motor retraining [10]. Functional neurology interventions focus on enhancing the brain’s ability to integrate sensory inputs and recalibrate motor outputs through specific exercises and manual therapies designed to stimulate proprioceptive, vestibular, and cognitive pathways [11,12]. Recent studies have highlighted that proprioceptive recalibration (through targeted stimulation of mechanoreceptors and joint proprioceptors) can positively modulate vestibular control and improve dynamic postural stability [13,14]. Additionally, cortical arousal modulation, a key aspect of functional neurology, has been linked to improved sensorimotor performance, suggesting that enhancing cortical excitability may facilitate more effective vestibular processing and VOR suppression [15].

Experimental evidence supports the view that rehabilitation techniques promoting proprioceptive recalibration lead to measurable improvements in vestibular gain, gaze stability, and balance control, particularly in populations with vestibular hypofunction or postural instability [16,17,18]. Furthermore, interventions that stimulate cortical arousal and improve cognitive–motor integration have been shown to enhance neuromuscular performance and reduce compensatory cognitive demands associated with vestibular dysfunction [17]. In particular, individualized proprioceptive stimulation, cortical arousal modulation, and sensorimotor retraining aimed at enhancing vestibular–ocular integration represent functional neurology practices that have not been extensively explored in studies addressing VOR dysfunctions. Despite these promising findings, there remains a gap in the literature regarding the acute psychophysiological and neuromuscular responses following functional neurology interventions specifically targeting VOR cancellation dysfunctions. Most existing studies focus on long-term vestibular rehabilitation outcomes or general vestibular function without isolating the effects on central VOR suppression mechanisms.

Therefore, the present study aimed to analyze the psychophysiological and neuromuscular reflex modifications following a single functional neurology intervention in individuals presenting VOR cancellation dysfunction. In this study, the term “neuromuscular reflex” refers to the functional assessment of neuromuscular performance indicators—such as handgrip strength and tolerance to muscle fatigue under vestibular stimulation—rather than the direct measurement of specific monosynaptic reflex arcs. These assessments serve as proxies for evaluating neuromuscular integration efficiency in the context of VOR cancellation performance. By examining acute changes in tolerance to VOR cancellation stimuli, pressure pain threshold, cortical arousal, handgrip strength, and cardiovascular parameters, we sought to provide novel insights into the immediate benefits of functional neurology interventions. We hypothesized that the functional neurology intervention would lead to significant improvements in VOR cancellation performance and related psychophysiological outcomes, reflecting enhanced neuroplastic adaptations, improved sensory integration, and optimized neuromuscular function.

## 2. Materials and Methods

### 2.1. Participant

We analyzed 66 healthy participants divided into an experimental group (*n* = 48; 22 females, 26 males; mean age 28.1 ± 7.8 years; height 170.6 ± 8.8 cm; weight 67.5 ± 12.8 kg; BMI 23.0 ± 3.0) and a control group of 18 participants (9 females, 9 males; mean age 28.6 ± 7.0 years; height 171.9 ± 8.6 cm; weight 68.2 ± 9.1 kg; BMI 23.1 ± 2.5). Inclusion criteria specified that all participants were free of medication, and without any neurological disorders. Eligibility was limited to healthy individuals aged 18 to 50 years, free from neurological disorders, musculoskeletal impairments affecting balance, uncorrected visual impairments, chronic illnesses, or any condition that could interfere with vestibular or motor performance. Participants were required to abstain from intense physical activity for 24 h, alcohol for 48 h, and any stimulants or central nervous system depressants such as caffeine or sedative medications for 24 h prior to the study. Additionally, a fasting period of two hours before participation was mandated. The study was conducted in full compliance with the Helsinki Declaration and received approval from the Bioethics Committee of the University (approval code: 2024-736, date: 10 June 2024).

### 2.2. Methodology

Our methodology involved assessing each participant’s response at four distinct times to understand the influence of the functional neurology intervention on their psychophysiological and muscular performance:

1. Baseline Measurement: Initial evaluations were performed to establish baseline data for each participant’s psychophysiological state and physical capabilities.
2. Pre-Intervention Post-Indicator Muscle Failure: Following baseline measurements, we administered a test to induce failure in the anterior deltoid muscle by exposing it to semicircular canal stimuli, documenting the muscle’s natural neurological response to stress before any treatment.
3. Post-Functional Neurology Treatment: After inducing muscle failure, participants underwent tailored functional neurology treatments designed to address neuromuscular imbalances and enhance neuroplasticity. Measurements post-treatment assessed immediate changes from the interventions.
4. Post-Intervention Post-Indicator Muscle Failure: Finally, we repeated the muscle failure test to evaluate the long-term impact of the treatment on muscle resilience and overall neuromuscular health.

The anterior deltoid muscle was utilized as an indicator muscle to assess CNS responses to vestibular stimulation within the functional neurology protocol applied. This muscle was chosen due to its involvement in upper limb proprioceptive stabilization and its sensitivity to vestibulo-ocular integration mechanisms. Baseline muscle function was first confirmed. Subsequently, semicircular canal stimulation requiring VOR cancellation was applied, and failure to maintain anterior deltoid isometric contraction was interpreted as a neurophysiological marker of impaired CNS vestibular integration. This approach is based on the principle that dysfunctional sensory integration can provoke generalized motor inhibition observable in indicator muscles.

### 2.3. Environmental Conditions and Instruments

All assessments were conducted under controlled conditions with a temperature of 23.5 ± 0.9 °C and humidity of 41.2 ± 2.8%. The instruments used included the following:

- Body weight measurements: Conducted using a SECA scale, model 714, calibrated and positioned on a hard, level surface. Participants were measured without heavy clothing or footwear [18].
- Pressure pain threshold (PPT): Measured using a Wagner Instruments Inc. (Greenwich, CT, USA) FPK 60 model manual pressure algometer, applied vertically to specific muscle areas until pain onset was indicated by the participant [19].
- Cortical arousal: Assessed using the critical flicker fusion threshold (CFFT) from a Lafayette Instrument Control Unit Model 12021. The Lafayette Instrument Flicker Fusion Control Unit was employed to assess cortical arousal by progressively increasing the flicker frequency of a light stimulus until participants reported a steady visual perception, recording the critical flicker fusion threshold. Changes in CFFT values indicate variations in cortical arousal and processing efficiency [20].
- Isometric handgrip strength: Measured with a TKK 5402 dynamometer from Takei Scientific Instruments Co. Ltd. (Niigata, Japan) Participants were seated with their arm at a 90-degree angle during the test [21].
- Blood oxygen saturation and heart rate: Monitored using a Beurer medical PO 30 pulse oximeter to assess cardiovascular response during the intervention [22].
- VOR cancellation test: The participant is seated upright and instructed to maintain visual fixation on their right thumb, positioned directly in front of them. The participant then actively moves their head horizontally from right to left at a moderate and controlled speed while continuing to track their thumb, allowing evaluation of their ability to suppress the vestibulo-ocular reflex. Semicircular canal stimulation was achieved through active head rotations at approximately 2 Hz along the horizontal axis, requiring continuous VOR cancellation while maintaining visual fixation.

The VOR cancellation tolerance assessment was performed following a standardized protocol to ensure measurement consistency. Participants were seated upright and instructed to fixate on their right thumb while performing controlled horizontal head movements. The evaluation endpoint—defined as the point of failure in maintaining isometric contraction of the anterior deltoid muscle—was objectively determined based on a visible loss of muscle function assessed through manual testing. Although complete assessor blinding was not feasible given the intervention structure, all evaluations were conducted by trained assessors who were independent from the intervention delivery team. Environmental conditions (temperature and humidity) and procedural instructions were strictly standardized for all sessions to minimize external influences and ensure replicability.

### 2.4. Functional Neurology Intervention

Participants received a tailored functional neurology treatment based on the distinct protocols of ^®^NeuroReEvolution (http://nre-therapy.com/ accessed on 10 April 2025), noted for its specialized approach. NeuroReEvolution^®^ is a proprietary intervention based on functional neurology principles, incorporating targeted proprioceptive stimulation and sensorimotor retraining to promote neuroplastic adaptations and optimize vestibular-ocular integration. The intervention aimed to reduce vestibular-related discomfort and to aid rehabilitation by promoting sensory reintegration, recalibrating vestibulo-ocular pathways, and enhancing balance recovery mechanisms. The treatment began with an in-depth clinical evaluation that included both oral and visual assessments, as well as targeted functional neurology tests on joints to identify specific neurological deficits in each participant. A practitioner, holding a Level III certification in the Functional Neurology Manual Muscle Test from ^®^NeuroReEvolution, utilized this detailed diagnostic information to address unique neurological imbalances in each participant, ensuring the therapy was customized and effective [23]. The core of the functional neurology approach focuses on enhancing proprioceptive responses, easing the sensitivity of trigger points, and employing a systemic approach to treatment using the blink reflex. Proprioceptive responses are automatic nervous system reactions to physical stimuli such as muscle stretching or tendon compression, crucial for maintaining posture, balance, and mobility. Our interventions aimed to correct abnormalities in these reflexes that could cause discomfort or restrict movement. The therapy involved precise identification and treatment of trigger points—specific muscle areas that, when stressed, can cause pain or tension in other parts of the body. By manually manipulating these points and applying targeted stimuli, the treatment worked to lessen their sensitivity, thereby recalibrating the nervous system’s responses to reduce discomfort and aid in rehabilitation. Embracing a holistic viewpoint, our therapy treated the body as an interconnected system, recognizing that a problem in one area could influence distant regions through the network of neural reflexes. This comprehensive approach aimed to address not only localized dysfunctions but also related conditions impacting the overall neuromuscular health of the individual [24].

### 2.5. Statistical Analysis

The statistical analysis was performed using IBM SPSS Statistics software, version 24.0. Data were initially assessed for normality for each variable at each measurement moment using the Kolmogorov–Smirnov test, which confirmed a normal distribution for all dependent variables (pressure pain threshold, hand strength, critical flicker fusion threshold, blood oxygen saturation, and heart rate) with *p*-values greater than 0.05. Statistical analyses incorporated VOR cancellation tolerance as a primary dependent variable, assessed across four measurement moments. Each psychophysiological and neuromuscular variable was measured across four distinct moments (baseline, post-indicator muscle failure, post-intervention, and post-second indicator muscle failure), allowing assessment of within-subject changes over time. Levene’s test further confirmed the homogeneity of variances across groups and measurement moments (all *p* > 0.05), validating the use of parametric analyses. An independent samples *t*-test was used to analyze differences between the age, height, and weight of the participants. A mixed ANOVA was conducted to evaluate interactions between group factors (experimental vs. control) and measurement moments (baseline, post-indicator muscle failure pre-intervention, post-functional neurology treatment, and post-indicator muscle failure post-intervention). Post hoc analyses with Bonferroni corrections were applied when significant interactions were identified, examining differences between and within groups at specific measurement moments. Partial eta squared (η^2^ partial) was computed to assess the magnitude of the observed effects. Effect sizes were interpreted according to standard thresholds: small (η^2^ = 0.01), medium (η^2^ = 0.06), and large (η^2^ = 0.14). Differences were considered statistically significant when *p* ≤ 0.05.

## 3. Results

Independent-samples *t*-tests indicated no significant differences between the control and experimental groups for age (t = 0.124, *p* = 0.902), height (t = 0.372, *p* = 0.712), or weight (t = −0.217, *p* = 0.829). The functional neurology intervention significantly increased the number of VOR cancellation stimuli tolerated until dysfunction in an indicator muscle (anterior deltoid) from 1.0 ± 0.0 to 129.0 ± 36.7 stimuli (F = 587.786, *p* < 0.001, ηp^2^ = 0.926). Conversely, the control group did not exhibit significant changes, with stimuli tolerance slightly decreasing from 1.1 ± 1.2 to 1.0 ± 1.1 (F = 0.059, *p* = 0.810, ηp^2^ = 0.003). A significant interaction between groups was also found (F = 568.421, *p* < 0.001, ηp^2^ = 0.910).

The mixed ANOVA analysis revealed significant interaction effects between group (experimental vs. control) and measurement moments for all analyzed variables (PPT, hand strength, CFFT, blood oxygen saturation, and heart rate). Post hoc analyses with Bonferroni adjustments indicated significant differences between the experimental and control groups at the post-functional neurology treatment (*p* < 0.01) and post-indicator muscle failure (post-intervention) moments (*p* < 0.05), confirming the effectiveness of the functional neurology intervention. Specifically, the experimental group exhibited marked improvements across moments (all variables *p* < 0.05), whereas the control group showed stable measures with no significant changes (all *p* > 0.05) (Table 1). Post hoc analyses using Bonferroni correction showed significant changes across moments within the experimental group for all measured variables (*p* < 0.05), with stable values observed in the control group (*p* > 0.05).

Partial eta squared (η^2^) values were calculated to quantify the magnitude of differences between the experimental and control groups across the four measured moments. Results indicated a large effect size for VOR cancellation stimuli tolerance (η^2^ = 0.34), heart rate (η^2^ = 0.34), and PPT (η^2^ = 0.32), moderate to large effects for CFFT (η^2^ = 0.22) and blood oxygen saturation (η^2^ = 0.22), and a moderate effect for hand strength (η^2^ = 0.20). Interaction plots visually confirmed that while the control group remained stable, the experimental group exhibited clear and consistent improvements after the functional neurology intervention.

## 4. Discussion

The objective of this study was to analyze the effect of functional neurology intervention on the psychophysiological response and neuromuscular reflex performance of participants with VOR cancellation visual stimuli dysfunction. Our hypothesis proposed that functional neurology intervention would lead to significant improvements in VOR cancellation ability, enhancing both neuromuscular and psychophysiological responses. The results of our study support this hypothesis, demonstrating that participants exhibited marked improvements in VOR cancellation stimuli tolerance, with significant increases in the number of stimuli tolerated until dysfunction in the anterior deltoid muscle. Additionally, we observed significant improvements in other psychophysiological measures, such as pressure pain threshold and critical flicker fusion threshold, indicating enhanced neuromuscular and cognitive performance post-intervention.

This study evaluated several psychophysiological parameters, including cortical arousal (measured via critical flicker fusion threshold), pain perception (assessed through pressure pain threshold), and cardiovascular responses (blood oxygen saturation and heart rate), in addition to neuromuscular performance. The results of our study indicate significant changes in the PPT and stability in handgrip strength following the functional neurology intervention. The PPT showed a significant increase in post-intervention, demonstrating enhanced pain tolerance in participants. This improvement aligns with previous findings that suggest the effectiveness of targeted neurological exercises in increasing pain thresholds through enhanced neural pathways and neuroplasticity [25]. Along this line, other studies have shown that interventions such as neural mobilization and vestibular rehabilitation can effectively increase pain thresholds, thereby improving overall pain management [26,27], supporting our results on the efficacy of functional neurology approaches in pain management. In contrast, handgrip strength remained relatively unchanged throughout the study, with no significant differences observed between the pre- and post-intervention measurements. This stability in grip strength may suggest that the functional neurology interventions specifically targeted pain modulation and neuromuscular responses without significantly impacting overall muscle strength in this vestibular dysfunction. Previous research has also reported mixed results regarding the impact of similar interventions on muscle strength, indicating that while pain sensitivity may improve, muscle strength might require different or additional intervention strategies [28]. Regarding handgrip strength, although a statistically significant improvement was detected following the functional neurology intervention, the magnitude of change was moderate compared to baseline values. This suggests that the intervention may have enhanced neuromuscular coordination and motor unit recruitment efficiency rather than producing substantial gains in maximal muscular strength. Similar findings have been reported in studies where sensorimotor recalibration protocols led to improved fine motor control and muscle activation patterns without necessarily inducing hypertrophic or strength-specific adaptations [29]. Therefore, while the handgrip strength findings are promising, their clinical relevance likely pertains to functional neuromuscular integration rather than maximal force generation.

Regarding the critical flicker fusion threshold values, we showed a significant decrease in post-intervention, indicating an increase in cortical arousal and information processing efficiency [30]. This finding is consistent with previous research, which also reported a decrease in cortical arousal values following physical and cognitive stressors, suggesting heightened CNS arousal [31,32,33]. The observed improvements in CFFT values after the intervention, together with the enhanced tolerance to VOR cancellation stimuli, suggest a reduction in CNS cognitive load. Increased CFFT thresholds have been associated with heightened cortical arousal and improved information processing efficiency, as reported in previous studies [30,31]. These findings indicate that functional neurology interventions may enhance cortical efficiency by alleviating compensatory demands typically imposed by vestibular dysfunction [34,35]. Such improvements likely reflect more efficient integration of vestibular and proprioceptive inputs, resulting in optimized sensorimotor performance and reduced reliance on higher-order cortical compensation mechanisms. Similarly, studies on the impact of exercise on CNS fatigue have shown that reduced CFFT values are indicative of increased cortical arousal and improved cortical processing capabilities [34]. The increase in cortical arousal evaluated after the functional neurology intervention highlights how this technique, by addressing vestibular system dysfunction, reduces the burden on the CNS. This reduction in CNS load leads to an improvement in cortical arousal, freeing cortical functions that were previously compromised by the dysfunction, a fact also reported in high-stress and high-eliciting environments [36,37]. This finding is supported by research indicating that resolving vestibular dysfunction can enhance cortical efficiency by alleviating the compensatory demands placed on the CNS. For instance, addressing vestibular dysfunction helps to stabilize gaze and posture, which in turn reduces the need for excessive cortical processing and compensatory mechanisms. This alleviation of cortical load can enhance cognitive and motor functions that were previously impaired [38].

Regarding blood oxygen saturation, following the measurement taken after the second application of VOR cancellation stimulation post-intervention, improvements were maintained compared to baseline values, where dysfunction occurred due to the VOR cancellation stimulus. This decrease can be attributed to the increased cognitive resource consumption of the CNS as it attempts to compensate for the vestibular dysfunction. Studies have shown that vestibular dysfunction can lead to increased cognitive load, which in turn can impact physiological parameters such as oxygen saturation [39]. The body’s compensatory mechanisms in response to vestibular disturbances often require significant cognitive effort, which may reduce the efficiency of other bodily functions, including oxygen utilization. The increased cognitive demand can lead to heightened sympathetic nervous system activity, affecting cardiovascular and respiratory functions [40]. Specifically, the CNS’s attempt to maintain balance and spatial orientation during vestibular challenges can strain cognitive resources, leading to observable changes in oxygen saturation levels. However, we can observe that the total values of blood oxygen saturation remained within normal ranges throughout the four evaluation points. Similarly, heart rate did not show significant differences during the evaluation periods, highlighting the low cardiovascular load implicated by the functional neurology intervention. This suggests that functional neurology interventions can stabilize physiological parameters such as blood oxygen saturation and heart rate, even under conditions that might typically induce variability. The maintenance of these parameters within normal ranges indicates that the intervention did not place excessive stress on the cardiovascular system, which is a positive outcome for patients with vestibular dysfunction.

Functional neurology aims to improve neural efficiency and overall CNS performance by addressing specific dysfunctions, such as those in the vestibular system. By resolving these dysfunctions, the interventions reduce the compensatory demands placed on the CNS, which can otherwise lead to significant physiological strain. This reduction in strain is reflected in the stable heart rate and blood oxygen saturation levels observed in this study, aligning with previous research that emphasizes the importance of maintaining stable vital signs to ensure effective CNS function and overall health [40,41]. Maintaining stable cardiovascular parameters during interventions is crucial, as fluctuations in these measures can indicate increased physiological stress or inadequate adaptation to the therapeutic regimen. The findings from this study support the notion that functional neurology interventions can be safely administered without adverse effects on cardiovascular function, providing a stable environment for neural rehabilitation [42]. Furthermore, recent studies have highlighted the potential benefits of functional neurology interventions in athletic rehabilitation and neuromuscular recovery, demonstrating their role in optimizing performance and reducing physiological burden in various populations [43,44]. Specifically, previous research has shown that these interventions contribute to improved muscular function and pain modulation by recalibrating neurological pathways and enhancing neuroplasticity, which further supports their application in vestibular dysfunction treatment [45]. Additionally, recent findings suggest that functional neurology techniques effectively reduce physiological stress markers, thereby improving autonomic regulation and neuromuscular coordination, which are essential for maintaining homeostasis and motor control in clinical and athletic settings [23,24]. The neuropsychophysiological effects of these interventions have been linked to improved semicircular canal function, further validating their relevance in the treatment of vestibular dysfunctions [46,47]. Future research should continue to explore the mechanisms behind these observations to further understand the full impact of functional neurology on both neural and cardiovascular health.

### 4.1. Study Limitations and Future Research Lines

This study has several limitations that need to be addressed in future research. Firstly, the sample size was relatively small, limiting the generalizability of the findings. Additionally, this study’s short duration may not fully capture the long-term effects of functional neurology interventions on VOR cancellation dysfunction. The reliance on self-reported measures for certain psychophysiological responses could introduce bias. Future studies should consider larger, randomized controlled trials with extended follow-up periods to validate and expand upon these findings. It is also recommended to incorporate more objective measurement tools to enhance the reliability of the data.

Another limitation of the present study is that, although efforts were made to minimize bias by separating evaluators from intervention providers, complete assessor blinding was not feasible due to the practical constraints of the intervention design. Future studies are encouraged to implement fully blinded or double-blind assessment protocols whenever possible to further enhance measurement reliability and reduce potential observer bias.

Future research should aim to explore the underlying mechanisms of functional neurology interventions in greater detail, particularly focusing on neuroplasticity and its role in VOR cancellation. Investigating the specific neural pathways involved and how these interventions modulate them could provide valuable insights. Additionally, studies should examine the effectiveness of functional neurology across diverse populations and different types of vestibular dysfunctions. There is also a need for research on the integration of functional neurology with other therapeutic modalities, such as pharmacological treatments, to evaluate synergistic effects. Longitudinal studies that monitor the sustained impact of these interventions over time would also be beneficial.

Moreover, the present findings, obtained in healthy individuals, offer a foundational basis for the design of future studies involving larger cohorts and clinical populations, particularly patients with confirmed vestibular loss. The significant improvements observed in psychophysiological and neuromuscular parameters following a single functional neurology intervention suggest promising therapeutic potential. Future investigations should aim to validate these preliminary results in patients with vestibular disorders, examining long-term efficacy, optimizing intervention protocols, and exploring individualized treatment approaches tailored to specific types and severities of vestibular dysfunction.

### 4.2. Practical Applications of Functional Neurology

Functional neurology offers practical applications that can significantly benefit individuals with VOR cancellation dysfunction and other neurological disorders. This approach leverages targeted exercises and manual therapies to enhance neural pathways and improve overall brain function. For instance, vestibular rehabilitation, a core component of functional neurology, involves specific head and eye movement exercises that recalibrate the vestibulo-ocular reflex pathways. This can enhance the brain’s capacity to suppress inappropriate VOR responses, thereby improving balance, reducing dizziness, and restoring normal eye movements. Moreover, functional neurology’s holistic approach, which includes lifestyle modifications and stress management, supports comprehensive management of VOR-related disorders, offering a promising avenue for long-term relief and rehabilitation.

## 5. Conclusions

The results of this study demonstrate that a single functional neurology intervention significantly improved psychophysiological responses and neuromuscular reflex performance in participants with vestibulo-ocular reflex cancellation dysfunction. Specifically, the intervention led to marked enhancements in pain tolerance, cortical arousal, and neuromuscular performance, while maintaining stable cardiovascular parameters. These findings indicate that functional neurology represents an effective therapeutic approach for addressing vestibulo-ocular reflex cancellation dysfunction by reducing central nervous system load and promoting neurophysiological efficiency.

## Figures and Tables

**Table 1:** Results of the experimental and control groups on psychophysiological and physical performance measures.

Group	Variable	Baseline	Post-Indicator Muscle Failure (Pre-Intervention)	Post-Functional Neurology Treatment	Post-Indicator Muscle Failure (Post-Intervention)	ANOVA *p*-Value
Experimental	Pressure Pain Threshold (kgf)	27.49 ± 0.67	29.69 ± 0.38	30.11 ± 0.33	35.69 ± 0.60	0.029 *
	Hand Strength (N)	20.41 ± 0.72	21.84 ± 0.39	23.15 ± 0.45	26.56 ± 0.52	0.012 *
	Critical Flicker Fusion Threshold (Hz)	32.24 ± 0.45	33.97 ± 0.53	37.38 ± 0.40	38.32 ± 0.60	0.003 *
	Blood Oxygen Saturation (%)	32.15 ± 0.33	35.05 ± 0.78	37.38 ± 0.45	35.74 ± 0.64	0.018 *
	Heart Rate (bpm)	22.44 ± 0.32	25.26 ± 0.43	27.09 ± 0.46	28.56 ± 0.57	0.008 *
Control	Pressure Pain Threshold (kgf)	39.50 ± 0.76	39.83 ± 0.34	38.89 ± 0.44	38.60 ± 0.71	0.450
	Hand Strength (N)	25.05 ± 0.70	25.69 ± 0.34	25.91 ± 0.66	26.06 ± 0.69	0.252
	Critical Flicker Fusion Threshold (Hz)	26.42 ± 0.33	27.72 ± 0.46	27.76 ± 0.62	26.72 ± 0.74	0.531
	Blood Oxygen Saturation (%)	22.88 ± 0.58	22.79 ± 0.69	24.08 ± 0.51	21.93 ± 0.31	0.276
	Heart Rate (bpm)	19.92 ± 0.55	21.15 ± 0.75	20.36 ± 0.68	19.88 ± 0.41	0.254

Baseline: initial measurement under resting conditions before intervention; post-indicator muscle failure (pre-intervention): measurement after inducing VOR dysfunction through semicircular canal stimulation; post-functional neurology treatment: measurement after completing the functional neurology intervention; and post-indicator muscle failure (post-intervention): measurement after a second VOR stimulation following intervention to assess recovery. Results are presented as means ± standard deviations; * indicates statistically significant differences compared to baseline (*p* < 0.05).

## Data Availability

All data are presented in the manuscript.
